# Plasma NT pro-BNP, hs-CRP and big-ET levels at admission as prognostic markers of survival in hospitalized patients with dilated cardiomyopathy: a single-center cohort study

**DOI:** 10.1186/1471-2261-14-67

**Published:** 2014-05-11

**Authors:** Xiaoping Li, Chengzhi Chen, Feng Gan, Yang Wang, Ligang Ding, Wei Hua

**Affiliations:** 1 Cardiac Arrhythmia Center, State Key Laboratory of Cardiovascular Disease, Fuwai Hospital, National Center for Cardiovascular Diseases, Chinese Academy of Medical Sciences and Peking Union Medical College, Beijing 100037, China; 2 Department of Cardiology, Sichuan Academy of Medical Sciences and Sichuan Provincial People’s Hospital, Chengdu 610072, China; 3 Department of Cardiology, Liuyang People Hospital, Liuyang 421001, China; 4 Department of Cardiology, Beijing Aerospace General Hospital, Beijing 100037, China

**Keywords:** Dilated cardiomyopathy, NT pro-BNP, Hs-CRP, Big-endothelin, Prognosis

## Abstract

**Background:**

Circulating N-terminal pro-B-type natriuretic peptide (NT pro-BNP), high- sensitivity C-reactive protein (hs-CRP) and big endothelin (big-ET) have been shown to be increased in heart failure and to contribute to both hemodynamic deterioration and cardiovascular remodeling. Here, we examined the prognostic value of the three neurohormones at admission in a population of hospitalized patients with dilated cardiomyopathy (DCM).

**Methods and results:**

This cohort study was undertaken in 622 hospitalized patients with DCM in Fuwai Hospital from January 2005 to September 2011 (female 26.5%, 51.4 ± 14.6 years old). Standard demographics, echocardiography and routine blood samples were obtained shortly after admission. NT pro-BNP, hs-CRP and big-ET were measured, and their concentrations in relation to all-cause mortality were assessed through a mean follow-up of 2.6 ± 1.6 years. Kaplan-Meier curves showed that the all-cause mortality rates were higher in patients with NT pro-BNP > 2247 pmol/L compared to patients with NT pro-BNP < 2247 pmol/L (11.9% vs 34.8%, log-rank *χ*^2^ = 35.588, *P* < 0.001), in patients with hs-CRP > 3.90 mg/L compared to patients with hs-CRP < 3.90 mg/L (12.8% vs 33.6%, log-rank *χ*^2^ = 39.662, *P* < 0.001) and in patients with big-ET > 0.95 pmol/L compared to patients with big-ET <0.95 pmol/L (12.5% vs 31.0%, log-rank *χ*^2^ = 17.890, *P* < 0.001). High circulating concentrations of NT pro-BNP (HR 2.217, 95% CI 1.015-4.846, *P* = 0.046) and hs-CRP (HR 1.922, 95% CI 1.236-2.988, *P* = 0.004), but not big-ET, in addition to left atrial diameter and fasting blood glucose, were independent predictors of the outcome defined as all-cause mortality.

**Conclusions:**

In a large population of patients with DCM, the circulating concentrations of NT pro-BNP and hs-CRP, but not big-ET, were independent markers of all-cause mortality.

## Background

B-type natriuretic peptide (BNP) is a cardiac peptide that is secreted from membrane granules in the cardiac ventricles as a response to ventricular volume expansion and pressure overload [1,2]. In the family of natriuretic peptides, N-terminal pro-B-type natriuretic peptide (NT pro-BNP) is the best predictor of clinical outcome because it is larger and has a longer half-life than BNP, making its measurement easier [3]. BNP and NT pro- BNP have become valuable biomarkers for confirming the diagnosis of heart failure (HF) [4,5] and have been predictive of outcomes among ambulatory and hospitalized HF patients [6-8].

C-reactive protein (CRP) is a nonspecific biochemical marker of inflammation. Studies have shown a link between CRP and various cardiovascular diseases such as atherosclerosis [9-11], hypertension [12], and chronic HF [13-15]. Increased CRP levels have been recognized as an independent predictor of all-cause and cardiovascular mortality in cardiovascular disease and in the general population [9-15]. Although the serum concentrations of CRP are elevated in patients with heart failure, especially those with severe acute HF [13-16], clinical data regarding the prognostic value of CRP in patients with chronic heart failure have been sparse and inconsistent [13,16,17]. Standard clinical assays for CRP lack sensitivity, but high-sensitivity CRP (hs-CRP) assays are now available and provide a better way to determine the predictive value for the prognosis of patients with CHF.

Elevated plasma concentrations of endothelin (ET) and of its precursor (big-ET) have been measured in patients with chronic HF [18-20] and have also been significantly associated with clinical outcomes in HF patients [21-24]. As circulating ET has a very short half-life, it is not easy to detect its circulating levels; however, circulating levels of big-ET have a longer half-life and are stable and easy to detect.

Although there have been clinical data showing that NT pro-BNP, hs-CRP and big-ET were elevated and were prognostic markers of survival in patients with chronic HF [6-8,13,16,17,21-24], there have been limited data on the prognostic values of these three biomarkers in DCM patients. Therefore, in the present study, we sought to investigate the significance of NT pro-BNP, hs-CRP and big-ET levels in hospitalized patients with DCM and to test the hypothesis that the three biomarkers could be prognostic markers of all-cause mortality in these patients.

## Methods

### Patients and follow-up

A retrospective, observational cohort study of 622 DCM patients from 2005 January to 2011 September was conducted. The patients were admitted for decompensated heart failure and the physical signs of HF at Fuwai Hospital, and all patients were hospitalized during 2005 January to 2011 September. DCM was defined as systolic dysfunction (left ventricular [LV] ejection fraction <50%) with LV dilation [defined as left ventricular end-diastolic diameter (LVEDd) > 55 mm for males or LVEDd > 50 mm for females] in the absence of an apparent secondary cause of cardiomyopathy [25] (e.g., ischaemic heart disease based on coronary angiography, overt hyper- and hypothyroidism thyroid disease, alcohol-induced cardiomyopathy, congenital heart disease, left ventricle noncompaction, chronic anaemia (hemoglobin <60 g/L), peripartum cardiomyopathy, heart valvular disease, combined with systemic immune disease or blood pressure more than 160/100 mmHg at admission). The primary end point of the study was all-cause mortality, which was assessed for all patients through their medical records (patients’ hospital records, periodically examining the patients in the outpatient clinic) and medical follow-up calls with trained personnel from admission until July 31, 2012. The follow-up rate was 92.7% with a mean follow-up duration of 2.6 ± 1.6 years. The study protocol was approved by the Ethics Commission of Fuwai Hospital.

### Measurement of big-ET, hs-CRP and NT pro-BNP

Plasma NT pro-BNP was measured in the day next to the admission using a commercial enzyme immunoassay (Biomedica, Vienna, Austria). The inter-assay coefficient of variation was 8% and the intra-assay coefficient of variation was 6%, with a detection limit of 171 pmol/L. Serum hs-CRP was measured using the Particle Enhanced Immunoturbidimetric Assay (Orion Diagnostica, Finland), where the detection limit was less than 0.25 mg/L, the inter-assay coefficient of variation was ≤8.71% and the intra-assay coefficient of variation was ≤11.9. Plasma big-ET was measured without prior extraction with the use of a commercial enzyme immunoassay (Biomedica, Vienna, Austria). Cross-reactivity was less than 1% for ET-1, ET-2, and ET-3. The inter-assay and intra-assay coefficients of variation were less than 5% and the detection limit was 0.02 pmol/L.

### Statistical analysis

Continuous variables are expressed as the mean ± SD or as medians and interquartile ranges. Baseline characteristics between the categories of NT pro-BNP (below/above 2247 pmol/L), hs-CRP (below/above 3.90 mg/L) and big-ET (below/above 0.95 pmol/L) were compared using the chi-square test for categorical variables; for continuous variables, comparisons were performed using an independent sample *t*-test.

Receiver-operating characteristic (ROC) analysis was performed to determine the optimal prognostic concentrations of big-ET, hs-CRP and NT pro-BNP for the study outcomes. Kaplan-Meier survival curves were compared using the log-rank test. Cox proportional hazard models were used to estimate the univariate and multivariable hazards of all-cause mortality associated with each individual parameter. Based on clinical and statistical significance in the univariate Cox analysis, the following covariates were used in multivariable models: age, gender, history of diabetes mellitus and atrial fibrillation (AF), New York Heart Association (NYHA) functional class, disease duration, blood pressure, QRS duration, left ventricle (LV) and left atrium (LA) diameters, LVEF, fasting blood glucose, serum creatinine levels, NT pro-BNP, hs-CRP and big-ET. SPSS (Statistical Package for the Social Sciences) version 16.0 software (SPSS, Chicago, Illinois) was used for all statistical analyses. All tests were 2-sided and a *P* value < 0.05 was used to determine statistical significance.

## Results

### Value of NT pro-BNP, hs-CRP and big-ET in predicting all-cause mortality

To evaluate the value of NT pro-BNP, hs-CRP and big-ET in predicting all-cause mortality in 622 patients with DCM, the receiver operating characteristic (ROC) curve was drawn. The optimal cutoff concentrations of NT pro-BNP, hs-CRP and big-ET for the prediction of the outcome of interest were determined using ROC analysis. For big-ET, the optimal cutoff of 0.95 pmol/L corresponded to a specificity of 0.597 and a sensitivity of 0.679 (area under the curve (AUC): 0.665 ± 0.028 [SD], P < 0.0001). For hs-CRP, the optimal cutoff of 3.90 mg/L corresponded to a specificity of 0.666 and a sensitivity of 0.634 (AUC 0.680 ± 0.026, P < 0 .0001). For NT pro-BNP, the optimal cutoff of 2247 pmol/L corresponded to a specificity of 0.712 and a sensitivity of 0.616 (AUC 0.703 ± 0.028, *P* < 0 .0001).

### Characteristics of the study population

The study consisted of 622 patients with DCM. The mean age of the enrolled patients was 51.4 ± 14.6 years, and 26.5% were female. Compared to patients with an NT pro-BNP <2247 pmol/L, patients with an NT pro-BNP >2247 pmol/L were more likely to be in NYHA classes III and IV, have larger LV diameters, more depressed LV ejection fractions (LVEF) and more dilated RV and LA diameters; these patients were also more likely to be taking aspirin and beta-blockers but were less likely to be taking digoxin. Patients with higher NT pro-BNP also had significantly higher levels of blood urea nitrogen and creatinine, higher heart rates, lower blood pressure and longer QRS duration. Compared to patients with hs-CRP < 3.90 mg/L, patients with hs-CRP > 3.90 mg/L were more frequently in NYHA classes III and IV and had more a depressed LV ejection fraction and more dilated RV and LA diameter. Additionally, these patients were more likely to be on digoxin and less likely to be taking ACEIs and beta-blockers, and had significantly higher levels of NT pro-BNP, creatinine, and higher SBP and heart rates. Compared to patients with a concentration of big-ET-1 < 0.95 pmol/L, patients with a concentration of big-ET-1 > 0.95 pmol/L were more likely to have atrial fibrillation, to be in NYHA classes III and IV, to have more depressed LV ejection fractions and more dilated LV, right ventricle (RV) and left atrial (LA) diameters, more likely to be taking diuretics and digoxin, and had significantly higher levels of NT pro-BNP and creatinine (Table 1).

**Table 1:** **Patient characteristics categorized by plasma NT-pro-BNP levels**^**1**^

	**All of the patients (n = 560)**	**NTpro-BNP < 2247 pmol/mL (n = 362)**	**NT pro-BNP > 2247 pmol/mL (n = 198)**	***P *** **value**
Age (years)	51.0 ± 14.2	51.1 ± 13.4	51.0 ± 15.8	0.958
Female sex, n (%)	146 (26.1)	98 (27.1)	48 (24.2)	0.466
History				
Course	3 (0.5-7)	2 (0.35-7)	3 (0.6375-6)	0.909
Diabetes mellitus	83 (14.8)	58 (16.0)	25 (12.6)	0.280
Atrial fibrillation, n (%)	121 (21.6)	76 (21.0)	45 (22.7)	0.634
Smoker, n (%)^2^	265 (47.3)	169 (46.8)	96 (48.9)	0.625
Drinker, n (%)^2^	170 (30.4)	119 (32.9)	51 (26.0)	0.089
NYHA class III and IV, n (%)^3^	425 (75.9)	247 (68.2)	178 (89.9)	**<0.001**
Admission vital signs				
SBP (mm Hg)^3^	113.3 ± 17.7	116.0 ± 17.6	108.3 ± 16.7	**<0.001**
DBP (mm Hg)^3^	72.3 ± 12.6	73.3 ± 12.5	70.5 ± 12.5	**0.012**
Heart rate, beat/min	80.8 ± 17.1	79.1 ± 16.0	84.0 ± 18.6	**0.002**
Laboratory values at admission^2^				
Glucose (mmol/L)	5.53 ± 1.70	5.49 ± 1.43	5.62 ± 2.10	0.386
TG (mmol/L)^3^	1.58 ± 0.89	1.74 ± 0.99	1.27 ± 0.57	**<0.001**
TC (mmol/L)^3^	4.59 ± 1.11	4.78 ± 1.08	4.22 ± 1.07	**<0.001**
Creatinine (μmol/L)	91.9 ± 34.2	87.5 ± 26.5	100.0 ± 44.0	**<0.001**
BUN (μmol/L)^3^	8.02 ± 4.07	7.68 ± 3.90	8.63 ± 4.33	**0.010**
NT Pro-BNP (pmol/mL)^3^	1652.85 (837.375-2907.2)	1051.45 (700.7-1613.675)	3425.25 (2787.975-4713.125)	**<0.001**
Electrocardiogram data^4^				
QRS duration (ms)	119.6 ± 30.1	117.6 ± 28.3	123.4 ± 33.0	**0.029**
QT (ms)	408.0 ± 50.7	410.9 ± 49.6	402.6 ± 52.4	0.067
Echocardiography data^4^				
LV (mm)^3^	68.5 ± 9.5	67.1 ± 8.7	71.1 ± 10.3	**<0.001**
LVEF (mm)^3^	31.1 ± 8.4	32.6 ± 8.4	28.1 ± 7.6	**<0.001**
RV (mm)	24.2 ± 5.4	23.1 ± 4.9	26.2 ± 5.8	**<0.001**
LA (mm)^3^	44.5 ± 7.8	42.9 ± 7.7	47.4 ± 7.2	**<0.001**
Medicine at admission^2^				
Diuretics, n (%)	529 (94.5)	339 (94.4)	190 (97.4)	0.103
ACEI/ARB, n (%)^3^	473 (84.5)	315 (87.5)	158 (81.9)	0.073
Beta-blockers, n (%)	509 (90.9)	340 (94.4)	169 (87.1)	**0.003**
Digoxin, n (%)	449 (80.2)	280 (78.0)	169 (86.7)	**0.013**
Aspirin/anticoagulation n (%)	353 (63.0)	243 (67.5)	110 (56.4)	**0.010**
Spironolactone, n (%)	514 (91.8)	331 (91.9)	183 (93.8)	0.414

Note:

^1^Data are expressed as the mean ± SD, median (interquartile range) or percentage; *P* values are from an independent-samples *t*-test.

^2^Smoker was defined using standard questions about current smoking and lifetime smoking at least 100 cigarettes; drinkers who reported drinking 1 drink a day on average on days that they consumed alcohol during the previous year.

^3^*Abbreviations*: *NYHA* New York Heart Association, *SBP* systolic blood pressure *DBP* diastolic blood pressure, *BUN* blood urea nitrogen *TG* triglyceride, *TC* total cholesterol, *NT pro-BNP* N-terminal fragment pro-brain natriuretic peptide, *LV* left ventricle, *RV* right ventricle, *LA* left atrium, *LVEF* left ventricular ejection fraction, *ACEI* angiotensin-converting enzyme inhibitor, *ARB* angiotensin receptor blocker.

^4^Seven patients lacked echocardiography data, 9 patients lacked electrocardiogram data, 4 patient lacked fasting blood glucose, 1 patient lacked creatinine, 2 patients lacked BUN levels, 5 patients lacked triglyceride, total cholesterol, high density lipoprotein cholesterol, and low density lipoprotein cholesterol levels and 6 patients lacked medicines at admission.

The bold data indicated that *P<0.05*.

### Relation between NT pro-BNP, hs-CRP, big-ET levels and all-cause mortality

**Figure 1:**
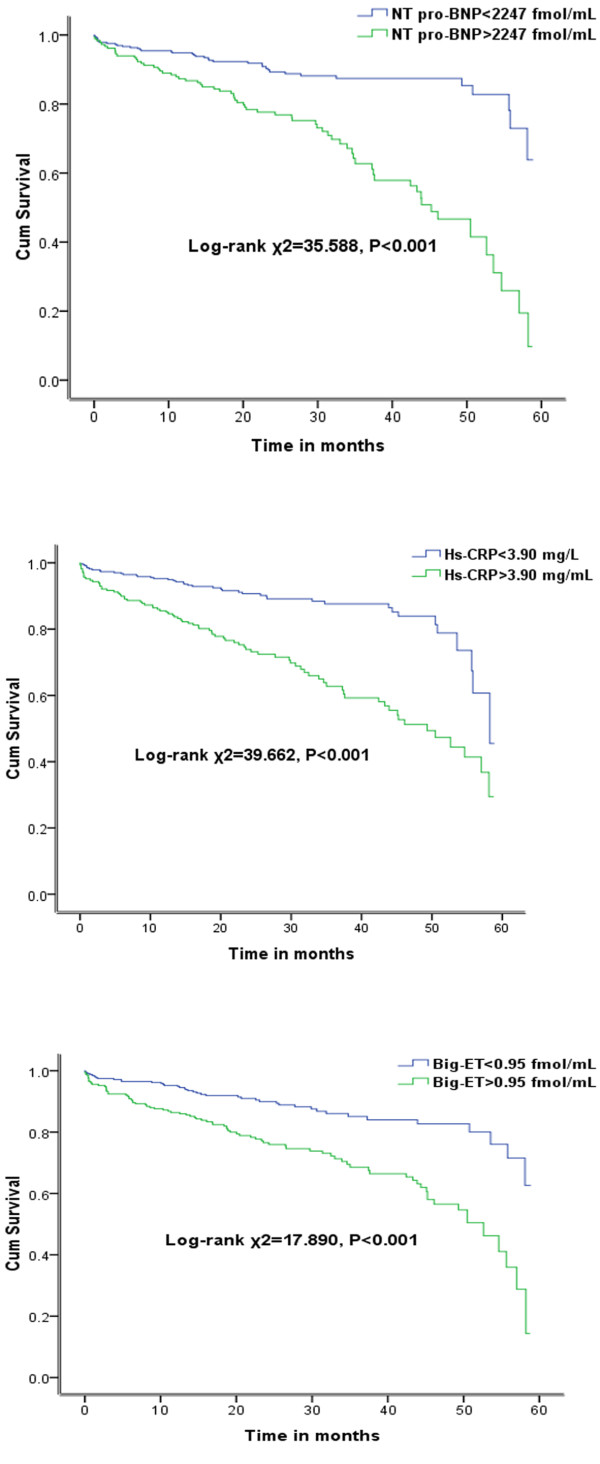
**Kaplan-Meier survival curves for patients with dilated cardiomyopathy: the upper panel: patients with NT pro-BNP < 2247 pmol/L and NT pro-BNP > 2247 pmol/L with DCM (log-rank *χ***^**2**^ **= 35.588, *P*** **< 0.001).** The middle panel: patients with hs-CRP < 3.90 mg/L and hs-CRP > 3.9 mg/L with DCM (log-rank *χ*^2^ = 39.622, *P* < 0.001). The lower panel: patients with big-ET < 0.95 pmol/L and big-ET > 0.95 pmol/L (log-rank *χ*^2^ = 17.890, *P* < 0.001).

Among the 622 patients studied, 131 (21.1%) died over a mean follow-up of 2.6 ± 1.6 years. At the mean follow-up of 2.6 years, Kaplan-Meier curves demonstrated that all-cause mortality was higher in patients with NT pro-BNP > 2247 pmol/L compared to the patients with NT pro-BNP < 2247 pmol/L (11.9% vs 34.8%, log-rank *χ*2 = 35.588, P < 0.001), that all-cause mortality rates were higher in patients with hs-CRP > 3.90 mg/L compared to the patients with hs-CRP < 3.90 mg/L (12.8% vs 33.6%, log-rank *χ*2 = 39.662, P < 0.001) and that all-cause mortality rates were higher in patients with big-ET > 0.95 pmol/L ompared to the patients with big-ET <0.95 pmol/L (12.5% vs 31.0%, log-rank *χ*2 = 17.890, P < 0.001) (Figure 1).

### Cox proportional hazard models

Univariate analyses indicated that age, history of essential hypertension and AF, NYHA functional classes, systolic blood pressure, QRS duration, LV and LA diameters, LVEF, circulating creatinine levels, NT pro-BNP, fasting blood glucose, serum creatinine levels, big-ET and hs-CRP were all predictors of all-cause mortality in DCM patients. After adjusting for age, gender, history of DM and AF, disease duration, creatinine levels, LV diameter and LVEF value, using either forward or backward selection, LA diameter, fasting blood glucose, hs-CRP > 3.90 mg/L and NT pro-BNP > 2247 pmol/L were the variables that remained in the model as the independent predictors of all-cause mortality. However, unlike hs-CRP > 3.90 mg/L and NT pro-BNP > 2247 pmol/L, big-ET > 0.95 pmol/L was not a predictor of death in the multivariate Cox analysis (Table 2).

**Table 2:** **Patient characteristics categorized by plasma hs-CRP or big-ET levels**^**1**^

	**All of the patients (n = 622)**	**hs-CRP < 3.91 mg/L (n = 375)**	**hs-CRP > 3.91 mg/L (n = 247)**	***P *** **value**	**Big ET < 0.95 pmol/mL (n = 335)**	**Big ET > 0.95 pmol/mL (n = 287)**	***P *** **value**
Age (years)	50.1 ± 14.3	50.1 ± 14.1	52.5 ± 14.2	0.051	51.2 ± 14.1	51.0 ± 14.5	0.846
Female sex, n (%)	160 (25.7)	102 (27.2)	58 (23.5)	0.299	88 (26.3)	72 (25.1)	0.737
History							
Course	3 (0.5-7)	2 (0.35-7)	3 (0.6-6)	0.170	3 (0.5-6)	2.5 (0.35-7)	0.628
Diabetes mellitus	92 (14.8)	57 (15.2)	35 (14.2)	0.723	45 (13.4)	47 (16.4)	0.303
Atrial fibrillation, n (%)	142 (22.8)	79 (21.1)	63 (25.5)	0.197	62 (18.5)	80 (27.9)	**0.006**
Smoker, n (%)^2^	294 (47.3)	172 (45.9)	122 (49.4)	0.396	159 (47.5)	135 (47.0)	0.953
Drinker, n (%)^2^	190 (30.5)	119 (31.7)	71 (28.7)	0.422	103 (30.7)	87 (30.3)	0.933
NYHA class III and IV, n (%)^3^	468 (75.2)	250 (66.7)	218 (88.3)	**<0.001**	217 (64.8)	251 (87.5)	**<0.001**
Admission vital signs							
SBP (mm Hg)^3^	113.2 ± 17.7	115.0 ± 31.1	118.7 ± 29.4	**0.002**	116.1 ± 17.6	109.7 ± 17.1	**<0.001**
DBP (mm Hg)^3^	72.2 ± 12.6	73.4 ± 12.1	70.4 ± 13.1	**0.004**	72.9 ± 12.2	71.4 ± 13.1	0.135
Heart rate, beat/min	80.5 ± 17.2	78.3 ± 16.2	83.9 ± 18.1	**<0.001**	78.7 ± 16.6	71.4 ± 13.1	**0.004**
Laboratory values at admission^4^							
Glucose (mmol/L)	5.52 ± 1.66	5.44 ± 1.39	5.64 ± 1.99	0.154	5.57 ± 1.65	5.46 ± 1.66	0.425
TG (mmol/L)^3^	1.58 ± 0.90	1.68 ± 1.00	1.41 ± 0.69	**<0.001**	1.74 ± 0.94	1.38 ± 0.81	**<0.001**
TC (mmol/L)^3^	4.57 ± 1.11	4.68 ± 1.02	4.40 ± 1.22	**0.004**	4.84 ± 1.06	4.25 ± 1.09	**<0.001**
Creatinine (μmol/L)	92.3 ± 34.7	88.2 ± 29.9	98.4 ± 40.3	**0.001**	89.0 ± 28.5	96.1 ± 40.6	**0.011**
BUN (μmol/L)^3^	8.02 ± 4.09	7.89 ± 4.21	8.22 ± 3.89	0.328	7.96 ± 4.10	8.10 ± 4.07	0.666
NT Pro-BNP (pmol/mL)^3^	1652.85 (837.375-2907.2)	1367 (736.75-2259.4)	2281.5 (1218.1-3589.4)	**<0.001**	1089 (714.675-1949.1)	2529.05 (1439.1-3608.475)	**<0.001**
Electrocardiogram data^2^							
QRS duration (ms)	119.2 ± 30.4	119.6 ± 31.1	118.7 ± 29.4	0.720	119.4 ± 30.2	119.0 ± 30.7	0.883
QT (ms)	407.7 ± 51.0	411.9 ± 50.5	401.2 ± 51.1	**0.011**	413.9 ± 49.6	400.4 ± 51.7	**0.001**
Echocardiography data^4^							
LV (mm)^3^	68.3 ± 9.5	67.8 ± 9.5	69.3 ± 9.4	0.055	67.3 ± 8.9	69.5 ± 10.1	**0.005**
LVEF (mm)^3^	31.2 ± 8.5	32.2 ± 8.7	29.8 ± 7.9	**0.001**	33.0 ± 8.5	29.2 ± 7.9	**<0.001**
RV (mm)	24.2 ± 5.5	23.3 ± 5.0	25.5 ± 5.9	**<0.001**	22.6 ± 4.2	26.0 ± 6.1	**<0.001**
LA (mm)^3^	44.4 ± 7.8	43.1 ± 7.6	46.5 ± 7.8	**<0.001**	42.3 ± 7.2	46.9 ± 7.8	**<0.001**
Medicine at admission^2^							
Diuretics, n (%)	589 (94.7)	352 (93.9)	237 (96.0)	0.080	314 (93.7)	275 (95.8)	**0.048**
ACEI/ARB, n (%)^3^	522 (83.9)	330 (88.0)	192 (77.7)	**0.003**	289 (86.3)	233 (81.2)	0.126
Beta-blockers, n (%)	564 (90.7)	349 (93.1)	215 (87.0)	**0.038**	306 (91.3)	258 (89.9)	0.857
Digoxin, n (%)	497 (79.9)	287 (76.5)	210 (85.0)	**0.004**	259 (77.3)	238 (82.9)	**0.038**
Aspirin/anticoagulation n (%)	393 (63.2)	237 (63.2)	156 (63.2)	0.868	211 (63.0)	182 (63.4)	0.725
Spironolactone, n (%)	572 (92.0)	347 (92.5)	225 (91.1)	0.837	306 (91.3)	266 (92.7)	0.193

Note:

^1^Data are expressed as the mean ± SD, median (interquartile range) or percentage; *P* values are from an independent-samples *t*-test.

^2^Smoker was defined using standard questions about current smoking and lifetime smoking at least 100 cigarettes; drinkers who reported drinking 1 drink a day on average on days that they consumed alcohol during the previous year.

^3^*Abbreviations: NYHA* New York Heart Association, *SBP* systolic blood pressure, *DBP* diastolic blood pressure, *BUN* blood urea nitrogen, *TG* triglycerides, *TC* total cholesterol, *NT pro-BNP* N-terminal fragment pro-brain natriuretic peptide, *LV* left ventricle, *RV* right ventricle, *LA* left atrium, *LVEF* left ventricular ejection fraction, *ACEI* angiotensin-converting enzyme inhibitor, *ARB* angiotensin receptor blocker.

^4^Ten patients lacked echocardiography data, 9 patients lacked electrocardiogram data, 4 patient lacked fasting blood glucose, 62 patients lacked NT-pro-BNP levels, 1 patient lacked creatinine, 2 patients lacked BUN levels, 5 patients lacked triglyceride, total cholesterol, high density lipoprotein cholesterol, and low density lipoprotein cholesterol, 62 patients lacked NT pro-BNP levels, and 6 patients lacked medicines at admission.

The bold data indicated that *P<0.05*.

## Discussion

In this study, we investigated the associations between circulating levels of NT pro-BNP, hs-CRP and big-ET and all-cause mortality in patients with DCM. Our major new finding suggests that at admission, plasma NT pro-BNP and hs-CRP, but not big-ET, were strong predictors of all-cause mortality in DCM patients, and this association was independent of traditional risk factors for adverse outcomes in HF and DCM such as age, LV diameter, NYHA functional class, and the LVEF.

Concentrations of NT pro-BNP are related to LV filling pressures and wall stress [26]. B-type natriuretic peptide levels have been shown to be elevated in patients with symptomatic LV dysfunction and correlate to LV filling pressure, NYHA classification and prognosis [27-29]. NT pro-BNP levels increase during left ventricular dysfunction and acute myocardial infarction, and serve to counteract mechanisms of heart failure through diuresis, natriuresis and antihypertensive effects [4-8]. Various timing of the natriuretic peptide measurements among hospitalized HF patients indicated that NT pro-BNP levels were associated with outcomes including mortality and morbidity [30-33]. There is also evidence that a BNP- or NT proBNP-guided strategy could improve morbidity related to chronic HF; a BNP-guided therapeutic strategy was superior to a clinically guided approach in NYHA functional class II to III patients who were considered optimally treated by chronic HF specialists [34,35]. There were limited data on the prognostic value of BNP or NT pro-BNP in DCM patients; the BNP or NT pro-BNP levels increased and were associated with cardiac events or mortality [36,37]. In the present study, NT pro-BNP was increased in patients in NYHA classes III and IV with depressed LVEF and larger dilated RV and LA diameters, and NT pro-BNP was one of the independent predictors of all-cause mortality in DCM patients.

Many studies have suggested that the role of CRP in cardiac disease is as an acute phase-reactant protein. CRP is mainly produced in the liver in response to interleukin-6 and plays many pathophysiological roles in the inflammatory process. Elevated levels of CRP have been observed in patients with HF [13-15], and activation of the immune response may play a role in heart failure through modifications in the renin-angiotensin-aldosterone and sympathetic systems [38,39]. There is evidence that chronic activation of the immune system such as monocyte-macrophage and lymphocyte activation exists in HF [38,39]. CRP may not only be a marker of chronic systemic inflammation but also may be directly involved in CHF. CRP can cause myocardial cell apoptosis, and thus ventricular damage or dysfunction [40]. In the present study, hs-CRP was increased in patients in NYHA classes III and IV with depressed LVEF, more dilated right ventricular and left atrial diameters. Patients with an hs-CRP >3.90 pmol/L also had higher all-cause mortality, and hs-CRP was one of the independent predictors of all-cause mortality in DCM patients.

As for big-ET, although many studies showed that big-ET levels were elevated and were prognostic markers of survival in patients with chronic heart failure [21-24], in the present study, big-ET was not one of the independent predictors of all-cause mortality in DCM patients.

The present study has several limitations; as with many studies of chronic disease, there may be variation in the length of the preclinical phase that influences the relationships between the three biomarkers of NT pro-BNP, hs-CRP, big-ET and death. The cohort in the present study included only patients with DCM who required hospitalization, and thus, the data described herein cannot be extrapolated to the entire DCM population. In addition, the medicines on admission should add some influence to the Cox multivariate analysis as without long-term medicine data. Finally, we measured the three biomarkers just at admission without the discharge values or more in the present study.

## Conclusion

In conclusion, in the present study, our findings suggest that hs-CRP > 3.90 mg/L and NT pro-BNP > 2247 pmol/L were associated with all-cause mortality in DCM patients and were some of the independent predictors of all-cause mortality after adjusting for the classic risk factors of HF in patients with DCM. However, big-ET > 0.95 pmol/L was not associated with higher all-cause mortality.

## Competing interests

The authors declare that they have no competing interests.

## Authors’ contributions

XL, CC and GF carried out the patient enrollment, data collection and follow-up. YW, XL and LD participated in the data collection and performed the statistical analyses. WH and XL conceived of the study, and participated in its design and coordination and helped to draft the manuscript. All authors read and approved the final manuscript.

## Pre-publication history

The pre-publication history for this paper can be accessed here:

http://www.biomedcentral.com/1471-2261/14/67/prepub
